# Diets, Fruit and Vegetable Intake, and Nutritional Status in Benin, Fiji, the Philippines, Sri Lanka, and Tanzania: Foreword

**DOI:** 10.1111/mcn.70163

**Published:** 2026-05-28

**Authors:** Deanna K. Olney, Mutinta Hambayi, Thushanthi Perera, Sonja Y. Hess

**Affiliations:** ^1^ International Food Policy Research Institute Washington District of Columbia USA; ^2^ World Food Programme Kigali Rwanda; ^3^ Department of Nutrition and Dietetics Wayamba University of Sri Lanka Makandura Sri Lanka; ^4^ Department of Nutrition Institute for Global Nutrition University of California Davis California USA

**Keywords:** diet, fruit, low‐ and middle‐income countries, nutrition, vegetables

## Abstract

Improving diets can improve nutrition and health outcomes. In this supplement, evidence from five low‐and‐middle‐income countries on the country specific dietary (including F&V) intake patterns, nutrition issues and evaluated solutions to improve diets across population groups is presented. Based on this evidence, the final paper offers perspectives and future priorities.

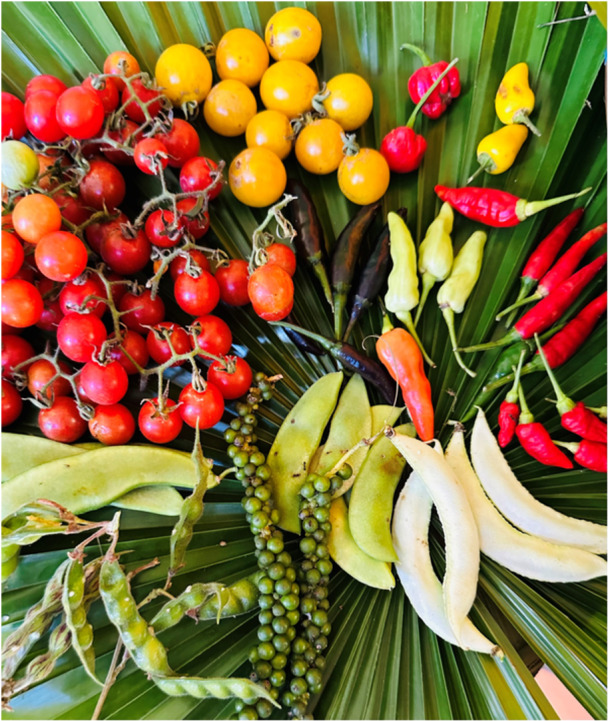

The double‐burden of malnutrition is increasing worldwide (Wells et al. 2020). Currently, about 372 million preschool‐aged children and 1.2 billion women of reproductive age are estimated to have at least one micronutrient deficiency (Stevens et al. 2022), and approximately 149 million children less than 5 years of age are estimated to be stunted and 45 million to be wasted (World Health Organization 2024a). At the same time overweight and obesity is affecting about 35 million children under 5 years of age (World Health Organization 2024b) and 1.11 billion women (Ng et al. 2025; Adult BMI Collaborators 2025) worldwide. These numbers are staggering and largely driven by poor diets which are the primary cause of all forms of malnutrition and the leading cause of disease worldwide (Global Panel on Agriculture and Food Systems for Nutrition 2016; Afshin et al. 2019; Diet Collaborators 2019; FAO, IFAD, UNICEF, WFP & WHO 2020).

Inadequate intake of fruit and vegetables is associated with an increased risk of non‐communicable disease (Li et al. 2014; Smith et al. 2022; Woodside et al. 2023). Improving diets, including increasing fruit and vegetable intake, can improve nutrition and health outcomes (Afshin et al. 2019; Diet Collaborators 2019). Yet, globally, fruit and vegetable intake is below recommended levels (Frank et al. 2019; Willett et al. 2019).

It is generally understood that the primary drivers of insufficient intake of fruit and vegetables boil down to issues around desirability, affordability, accessibility and availability which are often interconnected (Siegel 2019; Kaur 2023). For example, estimates suggest that 2–3 billion people cannot afford a healthy diet (Headey et al. 2024) with fruit and vegetables often being among the least affordable foods (FAO, IFAD, UNICEF, WFP & WHO 2020). Diverse and safe fruit and vegetables that are affordable are often inaccessible, especially to marginalized populations. This is in part due to availability issues that include inadequate production (Mason‐D'croz et al. 2019) and high post‐harvest losses among other issues (FAO 2014; Siegel 2019). In addition to constraints related to the affordability, accessibility and availability of fruits and vegetables, evidence also suggests issues related to the desirability of fruits and vegetables. For example, issues related to taste, convenience, inadequate storage space, appeal and competition of unhealthy foods and willingness of certain population groups like children and men to eat vegetables, among others, have been identified as barriers to the intake of fruit and vegetables (Kaur 2023). Solutions for improving diet quality, in part by increasing fruit and vegetable intake, will thus, need multifaceted and interconnected interventions.

Acknowledging these interrelated drivers, the CGIAR Research Initiative on Fruit and Vegetables for Sustainable Healthy Diets (FRESH) is using end‐to‐end approaches to increasing fruit and vegetable intake and improving related nutrition and health issues while also addressing environmental issues and improving livelihoods and the empowerment of women and youth (CGIAR 2024). The multi‐disciplinary and multi‐national collaborators of FRESH are working together to co‐design and evaluate holistic end‐to‐end approaches starting from fruit and vegetable intake and working back to address how the diet can be improved through influencing consumer behavior, improving food environments to encourage healthy food choices, and addressing post‐harvest, production, seed system and fruit and vegetable biodiversity challenges and related policies considering both the cultural context and environmental impact. These approaches recognize the complexity of food systems and the need for coordinated, multi‐disciplinary changes in many parts of the food system to improve dietary quality.

Although there is a general understanding of the issues driving inadequate intake of fruit and vegetables, there is still a paucity of data related to individual level dietary intake, dietary patterns, consumer preferences or desirability drivers, and food environments, especially in low‐ and middle‐income countries. Dietary patterns are difficult to change (de Ridder et al. 2017) but to initiate change, detailed individual‐level dietary data are needed to understand the extent of the dietary issues at the individual‐rather than household‐level and how they vary within households and across different population groups and regions. A better understanding of dietary patterns, current nutrition and health issues and their drivers are needed to inform the design of effective solutions to address these issues for different population groups in varying contexts.

In this supplement, we examine the state of the evidence related to diet (including a focus on fruit and vegetable intake) and nutrition issues in the four focal countries of FRESH (Benin, the Philippines, Sri Lanka and Tanzania). These countries have been chosen to represent different regions in Africa and Asia where inadequate intake of fruit and vegetables is common and where it is possible to address multiple food systems barriers to increasing the intake of fruit and vegetables. In addition, we examine available evidence for Fiji, a secondary focal country for FRESH, which, as an island nation, faces unique challenges with respect to the diet overall and fruit and vegetable intake.

The national‐level scoping reviews included in the present supplement provide insights on the issues at hand in each of the focal countries to help inform the design of interventions to increase fruit and vegetable intake (Amunga et al. 2025; Azupogo et al. 2025; Bliznashka et al. 2025; Koyratty et al. 2025; Smith et al. 2025), including those that will be implemented as part of FRESH's end‐to‐end approaches. An additional goal is to inform program implementers, policy makers and researchers alike to identify where there is a need to gather more comprehensive and longitudinal data related to dietary challenges, their drivers and solutions in these different contexts. In the final paper of the present supplement (Tharaney et al. 2025), we provide an overarching perspective on the issues and identified ways forward.

## Author Contributions

Deanna K. Olney drafted, and Mutinta Hambayi, Thushanthi Perera and Sonja Y. Hess edited the manuscript. All authors read and approved the final version of the paper.

## Conflicts of Interest

The authors declare no conflicts of interest.
